# Bioavailability of aspirin in fasted and fed states of a novel pharmaceutical lipid aspirin complex formulation

**DOI:** 10.1007/s11239-020-02051-5

**Published:** 2020-02-20

**Authors:** Dominick J. Angiolillo, Deepak L. Bhatt, Frank Lanza, Efthymios N. Deliargyris, Jayne Prats, Weihong Fan, Upendra Marathi

**Affiliations:** 1 Division of Cardiology, University of Florida College of Medicine, Jacksonville, 655 West 8th Street, Jacksonville, FL 32209 USA; 2 Brigham and Women’s Hospital Heart & Vascular Center, Harvard Medical School, Boston, MA USA; 3 Houston Institute for Clinical Research, Houston, TX USA; 4 PLx Pharma, Sparta, NJ USA; 5 Elysis LLC, Carlisle, MA USA; 6 7 Hills Pharma, Houston, TX USA

**Keywords:** Aspirin, Pharmacokinetic, Platelet, Bioavailability, Fasted, Fed

## Abstract

Dyspeptic symptoms are common with aspirin and clinicians frequently recommend that it be taken with food to reduce these side effects. However, food can interfere with absorption, especially with enteric-coated aspirin formulations. We evaluated whether food interferes with the bioavailability of a new, pharmaceutical lipid-aspirin complex (PL-ASA) liquid-filled capsule formulation. In this randomized, open label, crossover study, 20 healthy volunteers fasted for ≥ 10 h and then randomized as either “fasted”, receiving 650 mg of PL-ASA, or as “fed”, with a standard high-fat meal and 650 mg of PL-ASA 30 min later. After a washout of 7 days, participants crossed over to the other arm. The primary outcome was comparison of PK parameters of the stable aspirin metabolite salicylic acid (SA) between fasted and fed states. Mean age of participants was 36.8 years and 55% were male. The ratios for the fed to fasted states of the primary SA PK parameters of AUC_0−t_ and AUC_0−∞_ were 88.7% and 88.8% respectively, with 90% confidence intervals between 80 and 125%, which is consistent with FDA bioequivalence guidance. Mean peak SA concentration was about 22% lower and occurred about 1.5 h later in the fed state. Food had a modest effect on peak SA levels and the time required to reach them after PL-ASA administration, but did not impact the extent of exposure (AUC) compared with intake in a fasted state. These data demonstrate that PL-ASA may be co-administered with food without significant impact on aspirin bioavailability.

**Clinical Trial Registration:**http://www.clinicaltrials.gov Unique Identifier: NCT01244100

**Electronic supplementary material:**

The online version of this article (10.1007/s11239-020-02051-5) contains supplementary material, which is available to authorized users.

## Highlights

- Daily aspirin is frequently recommended to be taken with food in an effort to minimize dyspeptic side effects.
- Enteric coated aspirin suffers from erratic absorption and bioavailability that is further exacerbated when taken with food.
- The novel pharmaceutical lipid-aspirin complex (PL-ASA) liquid capsule formulation is an immediate release aspirin with predictable and consistent absorption and bioavailability that has been specifically designed to reduce aspirin’s GI toxicity.
- The results of the current study demonstrate that PL-ASA can be taken with food with only minimal impact on overall drug exposure and bioavailability.

## Introduction

Aspirin, or acetylsalicylic acid, is an irreversible inhibitor of the platelet cyclooxygenase (COX)-1 and COX-2 enzymes, causing a reduction in prostaglandin and direct prostaglandin derivatives such as thromboxane and producing several important downstream clinical effects. Aspirin is an effective antipyretic, analgesic and anti-inflammatory agent and has been clinically used for those conditions for over a century. However, the subsequent recognition that aspirin is also an effective inhibitor of platelet activation has resulted in its current use to be primarily for the prevention of cardiovascular events [1]. Although the role of aspirin for primary prevention is subject to controversy, aspirin is the cornerstone of treatment for secondary prevention in patients with atherosclerotic cardiovascular disease (ASCVD) [2–6]. The main side-effect of aspirin is related to its associated gastrointestinal (GI) toxicity, which can manifest as bleeding of varying severity and dyspeptic symptoms [7, 8]. Importantly, such GI side-effects are key contributors to poor adherence or discontinuation of aspirin therapy, which in turn can increase the risk of ischemic events in patients with ASCVD [9]. Clinicians frequently recommend that daily doses of aspirin be taken with food as a way to reduce dyspeptic symptoms [10]. However, food can interfere with aspirin absorption, especially with enteric-coated aspirin formulations, which are the most commonly used tablets in clinical practice [11]. Overall, these observations underscore the unmet need for aspirin formulations with a more favorable safety profile while maintaining pharmacologic efficacy.

A novel pharmaceutical lipid-aspirin complex (PL-ASA) liquid formulation was developed to mitigate disruption of the epithelial phospholipid layer of the gastric mucosa without delaying absorption [12–14]. Studies have shown that PL-ASA has similar bioavailability to immediate-release aspirin in fasted healthy volunteers and obese patients with diabetes [14, 15]. Moreover, PL-ASA significantly reduces the risk of acute gastric mucosal erosions and ulcers compared with immediate release aspirin [16]. The present study evaluated whether co-administration with food would interfere with the bioavailability of PL-ASA.

## Methods

### Study design and study population

**Fig. 1:**
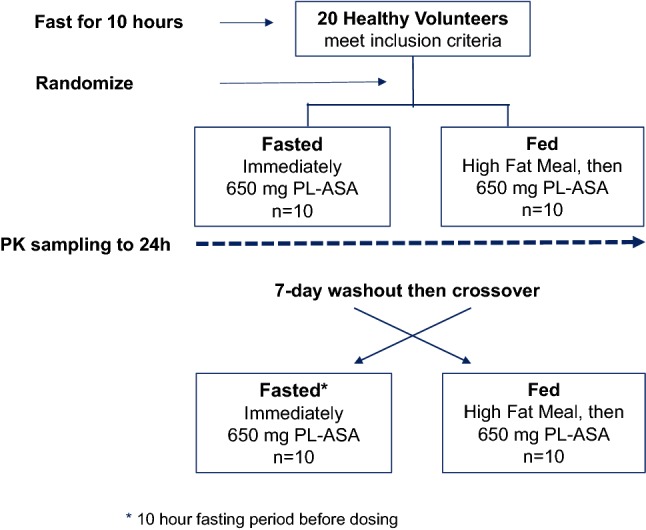
Study design

This was a single-center, randomized, active-controlled, open-label crossover study designed to assess fed vs. fasted pharmacokinetics (PK) of a single administration of PL-ASA (650-mg aspirin, administered as two 325-mg capsules) in 20 healthy volunteers from October to December 2010 (Clinicaltrials.gov identifier: NCT01244100). We selected 650 mg as the appropriate dose to test the impact of food since most patients take two 325 mg tablets when seeking acute pain relief or fever reduction. All subjects underwent screening procedures to determine eligibility for the study. In brief, healthy volunteers between 21 and 65 years of age who had not been exposed to an antiplatelet, anticoagulant or non-steroidal inflammatory agent in the previous 14 days were considered eligible for the study (See Online Supplement for specific study inclusion and exclusion criteria). Study subjects fasted for ≥ 10 h and underwent baseline evaluations prior to dosing. Eligible subjects were then randomized to 1 of the 2 treatment sequences, with ten subjects randomized to each sequence: 650 mg PL-ASA [fasted] or to 650 mg PL-ASA [fed]. If randomized to the fasted arm, subjects immediately received the single 650 mg dose of PL-ASA. If randomized to the fed arm, subjects first ate a standard high-fat, high-caloric meal and were dosed 30 min later. Each dose of PL-ASA was administered with 240 mL of water. A standard meal was provided for all subjects (fed or fasted) 4 h after administration of study drug and dinner was permitted immediately following the 10-h blood draw. After a washout period of 7 days each subject crossed over to the other arm and was again dosed with 650 mg of PL-ASA (i.e., subjects who received the first treatment in the fasted state, now received PL-ASA in the fed state and vice versa). Subjects had 6-mL blood samples drawn for PK analysis at the following time points after each study drug administration: within 1 h prior to administration, at 5, 10, 15, 20, 25, 30, 40, 50, 60, 75, and 90 min post administration; and at 2, 3, 4, 6, 8, 10, 12 and 24 h post administration. Laboratory assessments (hematology, blood chemistry) were performed at screening (Visit 1), prior to the second administration (Visit 3), and 24 h after each administration (Visits 2 and 4). The study design is illustrated in Fig. 1.

The protocol was approved by IntegReview Ethical Review Board (Number ORG0000689) and the study conducted at The Houston Institute for Clinical Research. Dr Angiolillo prepared the first draft of this manuscript and had full access to the analyzed data.

### PK analyses

Assessment of a food effect on PL-ASA bioavailability was determined by comparing PK parameters of salicylic acid (SA) after drug administration in the presence and absence of food. SA is the metabolite that is responsible for the analgesic and antipyretic effects of aspirin. SA is a far more stable metabolite compared with acetylsalicylic acid and is the analyte agreed upon by the Food and Drug Administration (FDA) for PK assessments of aspirin in this study. Specifically, acetylsalicylic acid is rapidly converted to SA by hydrolysis and first-pass metabolism making peak plasma acetylsalicylic acid concentrations extremely sensitive to minor variations in solid dosage form dissolution and disintegration. In contrast, plasma SA concentrations are predictable and relatively stable making it a more reliable analyte for PK assessments [15].

Accordingly, SA parameters were used for primary PK endpoints; while acetylsalicylic acid parameters were tested for secondary PK analysis. The primary PK assessments were made on AUC_0−t_, AUC_0−∞_, C_max_, t_max_, λz, t_½_, V_D_/F, CL/F and ratios of the least square means (LSM) of the log-transformed PK parameters of AUC_0−t_, AUC_0−∞_, C_max_ of SA in the presence or absence of food. Secondary PK assessments included similar parameters for acetylsalicylic acid in the presence and absence of food. A full list of PK parameters measured is provided in Online Table 2. Plasma samples were collected into sodium fluoride/potassium oxalate, frozen (− 80 °C) and analyzed by Medtox Laboratories (St. Paul, MN). Plasma SA and acetylsalicylic acid levels were determined by High Performance Liquid Chromatography with tandem Mass Spectrometry (LC- MS/MS) [15].

### Statistical methods

Full details regarding statistical methods are provided in the Online Supplement. Demographic and baseline characteristics were summarized using descriptive statistics: mean, standard deviation (SD), median, and range (minimum and maximum) for continuous variables and frequency and percentage of subjects for categorical variables. PK parameters of SA and acetylsalicylic acid were determined from individual plasma concentration data by non-compartmental analysis using the actual, exact sampling time in relation to dosing. PK parameters were summarized by the fed and fasted states. Mean, SD, coefficient of variance (CV), median and range were presented, and p-values were calculated based on the Wilcoxon Rank-Sum test. The statistical significance was assessed using a two-sided test at the 0.05 significance level.

Log-transformed parameters for AUC_0−t_, AUC_0−∞_, and C_max_ were calculated for each subject to determine the bioavailability ratio between fed and fasted states. The least-square means (LSM) of log-transformed PK parameter for both fed and fasted states were estimated by the use of a mixed-effects repeated measures Analysis of Variance (ANOVA) model. The model included sequence, period, fed/fasted state as fixed effects and subjects as a random effect. The exponentiality of LSM was called geometric mean. The ratio for each parameter was determined by dividing the geometric mean of fed state by the geometric mean of fasted state. To determine whether a meaningful food effect exists with PL-ASA, we referenced the FDA bioequivalence guidelines that propose that 90% confidence intervals (CI) of the ratio are between 80–125%.

## Results

A total of 24 subjects were screened for this study, of whom four did not meet study entry criteria. Thus, 20 subjects were enrolled, randomized, and treated with PL-ASA. All 20 treated subjects were 100% compliant with study drug administration and completed this study without protocol deviations. The baseline characteristics of the study population are summarized in Online Table 3. No adverse events were reported during the study. Vital signs and laboratory results were unremarkable.

**Fig. 2:**
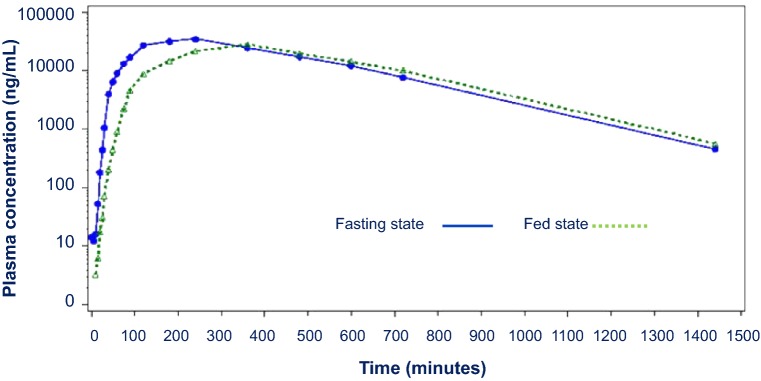
Mean plasma salicylic acid concentration versus time. Plasma concentrations (log-linear scale) for salicylic acid are depicted after a single 650 mg dose of PL-ASA in the presence (fed, dotted line) and absence of food (fasting, solid line)

Mean SA concentrations over time following a single 650 mg dose of PL-ASA in fed versus fasted states are graphically displayed in Fig. 2. Overall, the curves in the fasted and fed states were very similar, however, mean peak SA concentration was 28.1% higher in the fasted state and mean time to maximum SA concentration occurred about 1.5 h later in the fed state. Detailed listings of all SA parameters in both fed and fasted states are shown in Table 1. Most PK parameters in fed and fasted states were similar except for C_max_ that was significantly higher in the fasted state (p = 0.01), and t_max_ that was significantly higher in the fed state (p = 0.002).

**Table 1:** Summary of fed and fasted salicylic acid PK parameters after a single dose of 650 mg PL-ASA

PK parameter^a^	Fed N = 20			Fasted N = 20			P-value^b^
	Mean (SD)	CV (%)	Median (range)	Mean (SD)	CV (%)	Median (range)	
AUC_0−t_ ([µg × min] /mL)	14,945.7 (6436.2)	43.1	14,929.1 (7844.8–33,463.9)	16,521.8 (5958.7)	36.1	16,582.0 (7915.6–32,414.2)	0.3
AUC_0−∞_ ([µg × min] /mL)	15,202.9 (6723.0)	44.2	14,952.5 (8175.7–35,576.8)	16,791.0 (6167.9)	36.7	17,036.1 (8224.8–34,102.3)	0.3
C_max_ (µg/mL)	29.9 (8.6)	28.9	29.7 (17.9–55.3)	38.3 (9.9)	25.8	38.9 (22.2–57.8)	0.01
t_max_ (min)	283.5 (96.1)	33.9	360.0 (90.0–360.0)	180.0 (50.6)	28.1	180.0 (90.0–240.0)	0.002
λ_Z_ (1/min)	0.0048 (0.0009)	19.2	0.0050 (0.0023–0.0061)	0.0048 (0.0009)	18.9	0.0050 (0.0024–0.0060)	0.8
t_½_ (min)	152.9 (43.1)	28.4	137.5 (113.0–296.8)	152.9 (43.1)	28.2	139.2 (115.9–292.8)	0.8
CL/F (mL/min)	50.1 (19.2)	38.4	43.5 (18.3–79.5)	44.0 (16.7)	37.9	38.2 (19.1–79.0)	0.3
V_D_/F (mL)	10,400 (3128)	30.1	9880 (6086–15,433)	9148 (2498)	27.3	8093 (5644–13,824)	0.3

*AUC*_0−*t*_ area-under-the-curve; *AUC*_0−*∞*_ AUC_0−t_ extrapolated to infinity; *C*_*max*_ maximum plasma concentration; *CL/F* apparent clearance; *CV* coefficient of variation; *λ*_*z*_ terminal elimination rate constant; *μg* micrograms; *mg* milligrams; *min* minutes; *mL* milliliters; *n* number of subjects; *PK* pharmacokinetic; *PL-ASA* pharmaceutical lipid-aspirin complex; *SD* standard deviation; *t*_*max*_ time of peak drug concentration; *t*_*½*_ first-order elimination half-life; *V*_*D*_*/F* apparent volume of distribution

^a^N = 20 for all PK parameters

^b^P-value based on the Wilcoxon Rank-Sum test

Log-transformed AUC_0−t_, AUC_0-∞_ and C_max_ were used to calculate ratios of fed to fasted states as summarized in Table 2. To quantitively evaluate whether a food effect was present we applied the FDA guidance and determined that the 90% confidence intervals for the SA log-transformed AUC_0−t_ and AUC_0−∞_ ratios were indeed within the 80% to 125% range (82.2–95.8% and 82.2–96%, respectively) while the 90% CI for C_max_ was slightly outside that range (72.3–83.6%).

**Table 2:** Summary of ratios between fed and fasted states for log- transformed PK parameters of salicylic acid

PK parameter	N	LSM		Geometric mean		Ratio^a^ (%)	90% CI^b^	ANOVA p-value^c^
		Fed	Fasted	Fed	Fasted			
AUC_0−t_ (ng × min/mL)	20	9.53	9.65	13,772.6	15,521.3	88.7	(82.27, 95.8)	0.01
AUC_0−∞_ (ng × min/mL)	20	9.55	9.66	14,000.7	15,767.0	88.8	(82.2, 96.0)	0.02
C_max_ (ng/mL)	20	3.36	3.61	28.9	37.1	77.8	(72.3, 83.6)	< 0.0001

*ANOVA* analysis of variance; *AUC*_0−*t*_ area-under-the-curve; *AUC*_0−∞_ AUC_0−t_ extrapolated to infinity; *C*_*max*_ maximum plasma concentration; *CI* confidence interval; *LSM* least square mean; *mL* milliliters; *min* minutes; *N* number of subjects; *ng* nanograms; *PK* pharmacokinetic; *SD* standard deviation

^a^Ratio = 100% × geometric mean (fed) / geometric mean (fasted)

^b^90% Confidence interval on the ratio of fed and fasted

^c^p-value for the difference in the treatment estimates. Significant difference was defined as p-value < 0.05

**Fig. 3:**
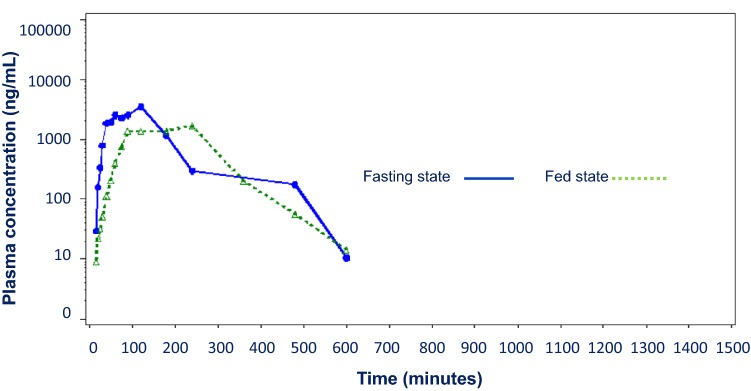
Mean plasma acetylsalicylic acid concentration versus time. Plasma concentrations (log-linear scale) for acetylsalicylic acid are depicted after a single 650 mg dose of PL-ASA in the presence (fed, dotted line) and absence of food (fasting, solid line)

Mean plasma acetylsalicylic acid concentrations over time in fed versus fasted states are graphically displayed in Fig. 3. Peak acetylsalicylic acid concentration was about 40% lower in the fed state and occurred about 1 h later compared with the fasted state. The summary of acetylsalicylic acid PK parameters in fed and fasted states is provided in Online Table 4. The slope of the log-linear elimination phase was not estimable in 10 subjects in the fed state and 14 subjects in the fasting state (18 unique subjects) due to an insufficient number of measurable ASA concentrations after the C_max_. While there were no significant differences noted between fed and fasted states in AUC_0−t_ and AUC_0−∞_ acetylsalicylic acid PK, there were significant differences noted in C_max_ (p = 0.003) and t_max_ (p = 0.01) suggesting a limited food effect on acetylsalicylic acid bioavailability. In aggregate, assessments of fed to fasted ratios for log-transformed acetylsalicylic acid parameters suggest a greater food effect compared to what was seen with SA.

## Discussion

The results of this study demonstrate that with food, even though the time required to reach maximum SA concentrations (T_max_) was significantly delayed and peak levels (C_max_) were also lower, there was a minimal effect on the overall exposure to SA (AUC), the primary aspirin metabolite, following PL-ASA administration. Since the efficacy of aspirin is believed to be related to overall exposure but not peak dose [17] the effects of food on peak SA levels and the time required to achieve them observed in our study are not considered to have clinical significance. Furthermore, the lower C_max_ observed with food after a 650 mg PL-ASA dose is still significantly higher than peak concentrations observed after 325-mg PL-ASA in a prior investigation in fasted healthy volunteers [15]. Similarly, any observed food effects on acetylsalicylic acid bioavailability are also not considered to be of clinical significance and are consistent with results in studies of uncoated aspirin [11, 18].

The differences in the median t_max_ values observed with food are consistent with the published results of similar analyses of immediate release and enteric-coated aspirin formations [11, 18]. In particular, in studies using immediate release aspirin tablets, acetylsalicylic acid levels were higher and peaked earlier in fasted subjects compared with fed subjects, indicating a decreased rate of absorption in the fed state [19]. However, overall bioavailability of the active metabolite, SA, demonstrated no food effect, as reflected in comparable salicylate levels in fed and fasted subjects [18]. Enteric-coated aspirin demonstrated a delayed and variable absorption rate relative to that of immediate release aspirin, particularly when administered in the fed state [11, 19, 20]. Enteric-coated aspirin absorption rates were shown to be significantly lowered by concurrent ingestion of food when compared to plain aspirin, with larger sized tablets being affected more significantly [21]. Current practice patterns suggest that enteric-coated aspirin is the dominant formulation in clinical practice and is frequently taken with food. It is therefore important to consider that enteric-coating itself, designed to hamper aspirin-induced gastrointestinal toxicity and improve aspirin adherence, may be responsible for delayed and impaired drug absorption, and hence contribute to variability in drug bioavailability. The implications are obvious since reduced aspirin bioavailability is associated with high rates of aspirin non-responsiveness that is linked to recurrent events among ASCVD patients [22].

Despite the available evidence of such food effects for commercially available aspirin formulations, it is notable that labels for consumers do not describe a food effect nor do they provide guidance on dose administration with respect to food consumption. In the current study, SA lag time (i.e., the time from dose administration to first appearance of salicylate in the blood) reported for PL-ASA at a 650-mg dose was 20.3 min ± 3.8 min in the fasted state and 65.3 min ± 39.4 min in the fed state. This is considerably lower than the reported lag times for enteric-coated aspirin at a dose of 648 mg of 162 min ± 48 min and 474 min ± 180 min for the fasted and fed states, respectively [20]. This data confirms that PL-ASA behaves as an immediate release formulation and that the food effect observed for PL-ASA is indeed less than that observed with enteric-coated aspirin.

## Study limitations

The present study was conducted in healthy volunteers who did not have an indication to be treated with aspirin. Moreover, the study was conducted using a dosing regimen of aspirin commonly used for anti-inflammatory effects. This approach is consistent with standard FDA guidance for bioequivalence studies in order to allow for pure pharmacologic comparisons without the risk of interaction or interference by underlying clinical conditions (i.e. healthy volunteers) and with dosing consistent with the clinical use on the proposed label which in the case of aspirin is for pain relief or fever reduction (i.e. 650 mg). The results from this study were included in the review and supported the subsequent FDA approval of the 325 mg dose [23]. The delay in reporting of these early data evaluating the food effect on the bioavailability of PL-ASA is the result of prioritization of studies focusing on antiplatelet activity (efficacy) and gastrointestinal toxicity (safety) following FDA approval. However, in light of the upcoming commercial availability of PL-ASA, investigators felt that publication of all prior data with PL-ASA, including information on fasted vs fed states on this novel, liquid-based formulation is important. Finally, since an 81 mg dose is also under development, it is important to note that the findings of our study with 650 mg cannot be extrapolated to the PL-ASA 81 mg dose, which warrants dedicated investigations.

## Conclusions

Food had a modest effect on peak SA levels and the time required to reach them after a single dose of 650 mg PL-ASA, but did not impact the extent of exposure (area under the curve) compared with intake on an empty stomach. These data demonstrate that PL-ASA may be taken with food without significant variability in absorption. Future investigations should focus on defining the impact of fasted vs fed states of PL-ASA compared with low dose enteric-coated aspirin, which is the current standard of care for secondary prevention in ASCVD patients.

## Electronic supplementary material

Below is the link to the electronic supplementary material.

Supplementary file1 (DOCX 49 kb)
