# Supplementary material for: Bioavailability of aspirin in fasted and fed states of a novel pharmaceutical lipid aspirin complex formulation
Source: J Thromb Thrombolysis. 2020 Feb 20;49(3):337–43. doi: 10.1007/s11239-020-02051-5 (PMC7145786; doi:10.1007/s11239-020-02051-5)
Supplement: Supplementary file 1 — Supplementary file1 (DOCX 49 kb) [file 11239_2020_2051_MOESM1_ESM.docx]

**Supplemental Appendix**

**Bioavailability of Aspirin in Fasted and Fed States of a Novel Pharmaceutical Lipid Aspirin Complex Formulation**

**Online Table 1.** Inclusion and Exclusion Criteria

**Statistical Methods**

**Online Table 2.** Primary and secondary PK and PD endpoints

**Online Table 3.** Baseline Characteristics

**Online Table 4.** Summary of Fed and Fasted Acetylsalicylic Acid PK Parameters after a Single Dose of 650 mg PL2200: PK Population

**Online Table 1. Inclusion and Exclusion Criteria**

| **Inclusion criteria** |
| --- |
| - Between 21 and 65 years of age, inclusive. - A normal physical examination. - Normal, or abnormal but not clinically significant, clinical laboratory test results during the screening visit, as assessed by the Investigator. - BMI between 20 and 32 kg/m^2^, inclusive. - If female and of childbearing potential, a negative serum pregnancy test and not be nursing. - If female and of child-bearing potential, agree to use adequate birth control for the duration of the study. - Able to understand and comply with study procedures. - Able and willing to provide written informed consent prior to any study procedures being performed. - Able to refrain from alcohol within 48 hours prior to and 24 hours after study drug administration. |
| **Exclusion criteria** |
| Subjects   - Abnormal screening/baseline laboratory parameters deemed clinically significant by the Investigator. - Use of any prescription medication within 14 days prior to study drug administration, other than hormone replacement therapy, thyroid replacement hormones, hyperlipidemic agents, anti-hypertensive medications, angiotensin-converting enzyme (ACE) inhibitors, or contraceptives. - Subject had taken any of the following medications within 14 days prior to study entry:   - NSAIDs or other medications for pain, including aspirin or aspirin-containing products   - Proton pump inhibitors including Prilosec® (omeprazole), Prevacid® (lansoprazole), Aciphex® (rabeprazole), Protonix® (pantoprazole), Nexium® (esomeprazole), or Zegerid® (omeprazole)   - H-2 blockers including Tagamet® (cimetidine), Zantac® (ranitidine), Axid® (nizatidine), or Pepcid® (famotidine)   - any anti-platelet agent, including Plavix® (clopidogrel), Ticlid® (ticlopidine), Pletal® (cilostazol), ReoPro® (abciximab), Integrilin® (eptifibatide), Aggrastat® (tirofiban), or Persantine® (dipyridamole)   - any anti-coagulant, including Coumadin® (warfarin), acenocoumarol, phenprocoumon, phenindione, heparin, Exanta® (ximelagatran), argatroban, lepirudin, hirudin, or bivalirudin - Use of an investigational agent within the 30 days previous to study entry. - Hypersensitivity or contraindications to aspirin, ibuprofen, or other NSAID. - History of stroke, myocardial infarction, or congestive heart failure. - Sensitivity to lecithin. - History of gastrointestinal problems, including ulcers, frequent indigestion, or heartburn. - History of asthma, other bronchospastic activity, polyps, or angioedema, other than resolved childhood asthma, thrombocytopenia, neutropenia, or bleeding disorder, kidney or liver disease, or diagnosed with chronic hypertension. - Previous coronary arterial bypass. - Previous non-trauma related hemorrhage. - Smoker at the time of screening. - History of alcoholism or drinking more than 1 alcoholic beverage per day at the time of screening. - Any other significant diagnosis or illness that the Investigator believed would interfere with the safety of the subject or the integrity of the data for the study. - Enrollment in another investigational trial at the time of screening. |

**Statistical Methods**

Standard statistical methods such as descriptive statistics, t-test, analysis of variance (ANOVA), and graphical displays were employed to analyze the data. Assumptions of normality and homogeneity of variance were tested using the Shapiro-Wilks test. If the distributional assumptions were not met, non-parametric techniques, such as Wilcoxon’s Rank-Sum test and Signed-Rank test were employed. Wilcoxon Signed Rank test p-values, along with the paired t-test p-values, were generated for all laboratory and vital signs presentations.

PK parameters were determined from the individual plasma concentration data of salicylic acid and acetylsalicylic acid by non-compartmental analysis using the actual, exact sampling times in relation to dosing. All PK parameters were analyzed for both the fed and fasted states. C_max_ and t_max_ were taken directly from the individual plasma concentrations. The λ_z_ was estimated by linear least squares regression with the logarithmic concentration data of the terminal part of the concentration-time curve. The AUC_0-t_ was calculated by the linear trapezoidal rule. Extrapolation of AUC_0-∞_ was made by dividing the last quantifiable concentration by the terminal elimination rate constant and adding this result to the AUC_0-t_. V_D_/F and CL/F were also calculated using area under the curve methods. Summary statistics for each PK parameter were calculated for each of the fed and fasted PK profiles.

Summary statistics consisted of frequencies and percentages of responses in each category for discrete measures and of counts, means, medians, standard deviations, 95% confidence intervals, and minimum and maximum values for continuous measures and are presented for the overall population and separately for the fed and fasted states.

For PK analyses, 90% confidence intervals were employed and any statistical significance was assessed using 2 one-sided tests at the 0.05 significance level. LSM was calculated using the exponentiation of the LSM from the analyses of the log-transformed AUC_0-t_, AUC_0-∞_ and C_max_. The 90% confidence intervals for the ratios were derived by exponentiation of the confidence intervals obtained for the difference between group LSM resulting from the analyses on the log-transformed AUC_0-t_, AUC_0-∞_ and C_max_. The ratios of LSM and 90% confidence intervals were expressed as a percentage of fed to fasted. The fed and fasted states were considered bioequivalent, indicating the absence of a food effect, if the 90% CIs of the geometric mean ratios for AUC_0-t_, AUC_0-∞_, and C_max_ for the fed and fasted states were within the 80% to 125% range recognized by the FDA to demonstrate bioequivalence.

**Online Table 2. Primary and secondary PK and PD endpoints**

|  | **Endpoints** |
| --- | --- |
| **Primary**  Salicylic acid | - AUC_0-t_ (area under the plasma concentration curve until last quantifiable value) - AUC_0-∞_ (AUC_0-t_ extrapolated to infinity) - C_max_ (observed maximum plasma concentration) - t_max_ (time of observed maximum drug concentration) - λ_z_ (terminal elimination rate constant) - t_½_ (terminal elimination half-life) - V_D_/F (apparent volume of distribution) - CL/F (apparent clearance) |
|  | - The ratios of the least square means (LSM) of the PK parameters of AUC_0-t_, AUC_0-∞_, and C_max_ of the primary metabolite salicylic acid in the fed state versus the fasted state were determined. |
| **Secondary**  Acetylsalicylic acid | - AUC_0-t_, AUC0-∞, C_max_, t_max_, λz, t_½_, V_D_/F, and CL/F of acetylsalicylic acid   The ratio of LSM of the fed state to the fasted state for the PK parameters of AUC_0-t_, AUC_0-∞_, and C_max_ of acetylsalicylic acid were used to assess the effect of food. |

**Online Table 3. Baseline Characteristics**

| **Baseline Characteristic** | **Overall (N=20)** |
| --- | --- |
| **Age (years)** |  |
| Mean (SD) | 36.8 (8.55) |
| Median | 35.0 |
| Minimum - Maximum | 22 – 56 |
| **Gender, n (%)** |  |
| Female | 9 (45.0) |
| Male | 11 (55.0) |
| **Race, n (%)** |  |
| White | 15 (75.0) |
| Black or African American | 3 (15.0) |
| Hispanic or Latino | 1 (5.0) |
| Asian | 1 (5.0) |
| **Height (inches)** |  |
| Mean (SD) | 68.4 (4.77) |
| Median | 70.0 |
| Minimum - Maximum | 61.0 – 77.0 |
| **Weight (pounds)** |  |
| Mean (SD) | 178.8 (40.84) |
| Median | 180.5 |
| Minimum - Maximum | 112.6 – 256.0 |
| **BMI (kg/m^2^)** |  |
| Mean (SD) | 26.6 (4.13) |
| Median | 27.9 |
| Minimum - Maximum | 20.0 – 31.3 |

BMI = body mass index, m=meters, kg=kilograms, n=number of patients, SD = standard deviation,

**Online Table 4. Summary of Fed and Fasted Acetylsalicylic Acid PK Parameters after a Single Dose of 650 mg PL-ASA: PK Population**

| **PK Parameter** | **Fed** | | | | | | **Fasted** | | | | | | |  |
| --- | --- | --- | --- | --- | --- | --- | --- | --- | --- | --- | --- | --- | --- | --- |
|  | **N*** | **Mean (SD)** | **CV**  **(%)** | **Median**  **(Range)** | | | **N*** | | **Mean**  **(SD)** | | **CV**  **(%)** | | **Median (Range)** | **P-Value^†^** |
| AUC_0-t_  ([ug x min]/mL) | 20 | 361.8  (157.4) | 43.5 | | 363.1  (121.3 – 887.2) | 20 | | 426.4  (131.4) | | 30.8 | | 425.9  (212.8 – 795.3) | | 0.07 |
| AUC_0-∞_  ([ug x min]/mL) | 10 | 394.4  (112.2) | 28.5 | | 384.1  (202.6 – 583.6) | 6 | | 410.2  (99.5) | | 24.3 | | 390.5  (297.0 – 561.0) | | 0.96 |
| C_max_ (µg/mL) | 20 | 3.1  (1.8) | 59.0 | | 2.7  (0.7 – 8.2) | 20 | | 5.4 (2.7) | | 48.8 | | 4.5  (2.1 – 11.0) | | 0.003 |
| t_max_ (min) | 20 | 171.8  (68.4) | 39.8 | | 180.0  (75.0 – 240.0) | 20 | | 109.3  (41.2) | | 37.7 | | 120.0  (40.0 – 180.0) | | 0.01 |
| λ_Z_ (l/min) | 10 | 0.0135  (0.0067) | 49.4 | | 0.013  (0.005 – 0.027) | 6 | | 0.0279  (0.0092) | | 32.8 | | 0.0292  (0.015 –0.041) | | 0.01 |
| t½ (min) | 10 | 68.3  (44.6) | 65.3 | | 52.2  (25.9 – 154.9) | 6 | | 27.7  (10.7) | | 38.8 | | 23.8  (16.9 – 46.5) | | 0.01 |
| CL/F  (mL/min) | 10 | 1790.3  (596.0) | 33.3 | | 1692.8  (1113.8 – 3207.9) | 6 | | 1661.9  (387.9) | | 23.3 | | 1685.9  (1158.7 – 2188.9) | | 1.0 |
| VD/F (mL) | 10 | 160368.4  (73643.1) | 45.9 | | 142771.4  (60960.8 –279388.3) | 6 | | 66903.1  (30150.5) | | 45.1 | | 60260.7  (33070.5-106169.8) | | 0.03 |

*N=20 for all PK parameters. The slope of the log-linear elimination phase was not estimable due to an insufficient number of measurable ASA concentrations after the C_max_ in 10 subjects in the fed state and 14 subjects in the fasting state (18 unique subjects), †P-value based on the Wilcoxon Rank-Sum test

AUC_0-t_ =area-under-the-curve**,** AUC_0-∞_ =AUC_0-t_ extrapolated to infinity, C_max_ =maximum plasma concentration, CL/F =apparent clearance, CV=coefficient of variation, λ_z_ =terminal elimination rate constant, μg=micrograms, mg=milligrams, min=minutes, mL=milliliters, n=number of patients, PK=pharmacokinetic, PL-ASA=pharmaceutical lipid-aspirin complex, SD= standard deviation. t_max_ =time of peak drug concentration, t_½_ =first-order elimination half-life, V_D_/F=apparent volume of distribution.
